# γ-Synuclein Interacts with Phospholipase Cβ2 to Modulate G Protein Activation

**DOI:** 10.1371/journal.pone.0041067

**Published:** 2012-08-08

**Authors:** Urszula Golebiewska, Yuanjian Guo, Narindra Khalikaprasad, Cassandra Zurawsky, V. Siddhartha Yerramilli, Suzanne Scarlata

**Affiliations:** 1 Department of Physiology & Biophysics, Stony Brook University, Stony Brook, New York, United States of America; 2 Department of Biological Sciences and Geology, Queensborough Community College, Bayside, New York, United States of America; University of South Florida College of Medicine, United States of America

## Abstract

Phospholipase Cβ2 (PLC β2) is activated by G proteins and generates calcium signals in cells. PLCβ2 is absent in normal breast tissue, but is highly expressed in breast tumors where its expression is correlated with the progression and migration of the tumor. This pattern of expression parallels the expression of the breast cancer specific gene protein 1 which is also known as γ-synuclein. The cellular function of γ-synuclein and the role it plays in proliferation are unknown. Here, we determined whether γ-synuclein can interact with PLCβ2 and affect its activity. Using co-immunprecitation and co-immunofluorescence, we find that in both benign and aggressive breast cancer cell lines γ-synuclein and PLCβ2 are associated. In solution, purified γ-synuclein binds to PLCβ2 with high affinity as measured by fluorescence methods. Protease digestion and mass spectrometry studies show that γ-synuclein binds to a site on the C-terminus of PLCβ2 that overlaps with the Gαq binding site. Additionally, γ-synuclein competes for Gαq association, but not for activators that bind to the N-terminus (i.e. Rac1 and Gβγ). Binding of γ-synuclein reduces the catalytic activity of PLCβ2 by mechanism that involves inhibition of product release without affecting membrane interactions. Since activated Gαq binds more strongly to PLCβ2 than γ-synuclein, addition of Gαq(GTPγS) to the γ-synuclein –PLCβ2 complex allows for relief of enzyme inhibition along with concomitant activation. We also find that Gβγ can reverse γ-synuclein inhibition without dissociating the γ-synuclein- PLCβ2- complex. These studies point to a role of γ-synuclein in promoting a more robust G protein activation of PLCβ2.

## Introduction

The synucleins are small (∼140 amino acid) proteins, that have a weak homology to 14.3.3 proteins (a typical member of the chaperone protein family (see [1], [2], [3]). The synucleins are considered to be “natively unfolded” [4] although recent work indicates that in cells α-synuclein folds into a dynamic tetramer [5], [6]. There are three members of the synuclein family, α, β and γ that are conserved and found throughout vertebrates. The cellular function(s) of synucleins have not yet been discovered. α-Synuclein, the most notable family member, is associated with neurodegenerative plaques [2].

Although γ-synuclein is found mostly in the peripheral nervous system and in pre-synaptic terminals, its over-expression is associated with cancer progression. γ-Synuclein was identified as the breast cancer specific gene protein 1 (BCSG1) ∼10 years ago by screening a breast cancer cDNA library [7]. γ-Synuclein is highly expressed in infiltrating breast cancer [8] but is undetectable in normal or benign breast lesions, and is partially expressed in ductal carcinomas. While the function of γ-synuclein is unknown, it is found in a wide variety of transformed cells and its overexpression leads to a significant increase in proliferation, motility, invasiveness and metastasis [8], [9].

Like γ-synuclein, phospholipase C β2 (PLCβ2) is absent in normal breast tissue, but is highly expressed in transformed tissue where its level of expression is directly related to tumor progression and migration [10], [11] presumably through its regulation by small G proteins [10], [11]. PLCβ2 is a member of a larger mammalian PLC family that catalyzes the hydrolysis of phosphatidylinositol 4,5 bisphosphate (PI(4,5)P_2_). Cleavage of PI(4,5)P_2_ generates the second messengers, diacylglycerol and 1,4,5 inositol trisphosphate (Ins(1,4,5)P_3_), which activate protein kinase C (PKC) and cause the release of Ca^2+^ from intracellular stores, respectively. All four isoforms of PLCβ are strongly activated by Gα_q_. Additionally, PLCβ2 and PLCβ3 are activated by Gßγ dimers that can potentially be released upon activation of all Gα families. It has also been found that PLCβ2 can be activated by members of the Rho family of monomeric G proteins with the strongest activation by Rac1, which is involved in the cytoskeletal rearrangements that accompany cell mobility [12].

PLCβ2 is a modular protein composed of an N-terminal pleckstrin homology (PH) domain, 4 EF hands, a catalytic domain, a C2 domain and a long C-terminal extension (see [13]). Crystallographic studies have indicated that Rac1 may promote enzyme activity by binding strongly to the PH domain and promoting membrane binding [14]. Alternately, Gβγ activates the enzyme by simultaneously interacting with both the PH and catalytic domains to change their domain orientation, while Gαq activates the enzyme through interactions with the C2 and C-terminal regions of the enzyme (see [15]). Even though PLCβ3 can be simultaneously activated by Gαq and Gβγ, this does not appear to occur for PLCβ2 [16].

Here, we have tested the idea that γ-synuclein interacts with PLCβ2 to promote cancerous phenotypes. We present data showing that they may associate in breast cancer cells and in solution. The binding of γ-synuclein to PLCβ2 results in inhibition of enzymatic activity that can be overcome by the addition of G protein subunits. This relief of γ-synuclein inhibition along with activation results in a more robust response of the enzyme.

## Materials and Methods

### Cell culture

MDA MB 231 cells were purchased from American Type Culture Collection (ATCC) and were cultured in Dulbecco's Minimum Essential Media (DMEM) supplemented with 10% Fetal Bovine Serum (FBS), 50 units/mL of penicillin and 50 μg/mL of streptomycin at 37°C and 5% CO_2_. MCF 10 A cells, also purchased from ATCC, were also cultured at 37°C and 5% CO_2_ in a medium that consists of 1∶1 mixture of Ham's F12 and DMEM media supplemented with 10% FBS, 50 units/mL of penicillin and 50 μg/mL of streptomycin, 10 μg/ml bovine insulin, 0.18 μg/ml hydrocortisone, and 20 ng/ml recombinant human epidermal growth factor.

### Immunofluorescence

Cells were fixed using 3.7% formaldehyde and permeabilized with 0.2% nonyl phenoxypolyethoxylethanol (NP40) and incubated with 0.2% NP40 in phosphate buffered saline (PBS) for 5 min and then blocked in PBS containing 4% goat serum for 1 h. The cells were then incubated with the primary antibody (anti-PLCβ2 (Santa Cruz Biochemicals, Inc.) and anti-γ-synuclein (Abcam, Inc.)) diluted to 1∶500 overnight at 4°C, followed by incubation with Alexa-labeled secondary antibody for 1.5 hours at room temperature. The cells were washed with tris buffered saline (TBS) buffer after the incubations. Images of the cells were obtained using Olympus Fluoview FV1000 laser scanning confocal microscope, and were analyzed using Olympus (Fluoview) software and Image J (NIH).

### Co-immunoprecipiation

MDA MB 231 cells were lysed with 500 ul of buffer containing 150 mM NaCl, 20 mM HEPES, 2 mM MgCl_2_, 5 mM 2-mercaptoethanol, 1 mM phenylmethylsulfonyl fluoride, 10 ug/ml leupeptin and 10ug/ml aprotinin. The lysate was then added to 20 µl of Protein A beads, which were incubated with 5 µl of rabbit anti-γ synuclein antibody overnight at 4°C, and the mixture was gently rotated for 4 hours at 4°C. The unbound proteins were separated from the beads, which were then washed twice with the lysis buffer. The bound proteins were then eluted from the beads in sample buffer at 95°C for 3 minutes and were loaded onto a 12% PAGE gel along with an equal volume of the unbound proteins for SDS-PAGE. After western transfer to polyvinylidene difluoride membranes, the membranes were blotted with anti- γ-synuclein and anti-PLCβ2 antibodies.

### Protein expression and purification

Human γ-synuclein was expressed and purified using an identical procedure as described for α-synuclein [17] with minor modifications. Gβ_1_ and His_6_-Gγ_2_ were co-expressed with Gαq in *Sf*9 cells and the heterotrimer was purified on a Ni^2+^-NTA column, and then dissociated by activation with GTPγS [18]. The purity of proteins was assessed by SDS-PAGE electrophoresis and western blotting. Concentrations were determined by a Bradford assay (BioRad) or on SDS-PAGE gels with known concentrations of BSA for reference. His_6_-PLCβ2 was expressed in Sf9 cells using a baculovirus system with minor modifications [17]. A C-terminal truncation mutant of PLCβ2 used for some of the control studies is a chimera of PLCβ2 and PLCδ1 described in [19]. Rac1 was a generous gift from Dr. Nicolas Nassar (Univ. Cincinnati) and its integrity was verified by SDS-PAGE electrophoresis and mass spectrometry and was prenylated using a kit from Uniprot, Inc.

### Digestion studies

µ-Calpain digestion was carried out by adding µ-calpain in 800 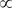
M calcium to pre-incubated PLCß2- γ-synuclein complexes, at 100 nM each, for 25°C for 20 minutes. The amount of digestion was assessed by western blot analysis using anti-PLCβ2 (Santa Cruz Biochemicals), or by assessing the loss in enzyme activity as determined by the ability of 20–50 nM enzyme to hydrolyze [^3^H]PI(4,5)P_2_ dispersed on 2 mM sonicated membranes composed of phosphatidyl serine: phosphatidyl ethanolamine: phosphatidyl inositol 4,5 bisphosphate (PS: PE: PI(4,5)P_2_) at a 2∶1∶0.5 molar ratio as described previously [20].

### Mass spectrometry

PLCβ2 bands alone or subjected to calpain digestion were isolated on SDS PAGE electrophoresis. The bands were cut and removed, digested by trypsin and the peptides were analyzed by LC/MS/MS on a Thermo LTQ XL at the Proteomics Center at Stony Brook University.

### Enzyme Activity Studies

Measurements of PI(4,5)P_2_ hydrolysis by PLC enzymes were carried out by doing small, unilamellar vesicles composed of 1-palmitoyl -2 –oleoyl phosphatidylethanolamine (PE): 1-palmitoyl-2-oleoyl phosphatidyl serine (PS) :PI(4,5)P_2_ at a 66∶32∶2 molar ratio with enough [^3^H]PI(4,5)P_2_ to achieve reliable signal(see [21]).

### Fluorescence labeling and titrations

Proteins were labeled on ice with the thiol-reactive probe7-diethylamino-3-(4′-maleimidylphenyl)-4-methylcoumarin (CPM) or Dabcyl at a probe:protein ratio of 4∶1. The reaction was stopped after 60 minutes by adding 10 mM DTT and the protein was purified either by extensive dialysis or using a PD G-25 spin trap column (GE Healthcare).

Fluorescence measurements were performed on an ISS spectrofluorometer (Champaign, IL) using 3 mm quartz cuvettes. Peptide and protein stocks were diluted into 20 mM Hepes (pH 7.2), 160 mM NaCl, 1 mM DTT. The emission spectrum of CPM-labeled protein was measured from 400 to 550 nm (λex = 380 nm). The background spectra of unlabeled protein or peptide were subtracted from each spectrum along the titration curve. All of the spectra were corrected for the 10–12% dilution that occurred during the titration.

## Results

### γ-Synuclein and PLCβ2 associate in breast cancer cells

**Figure 1:**
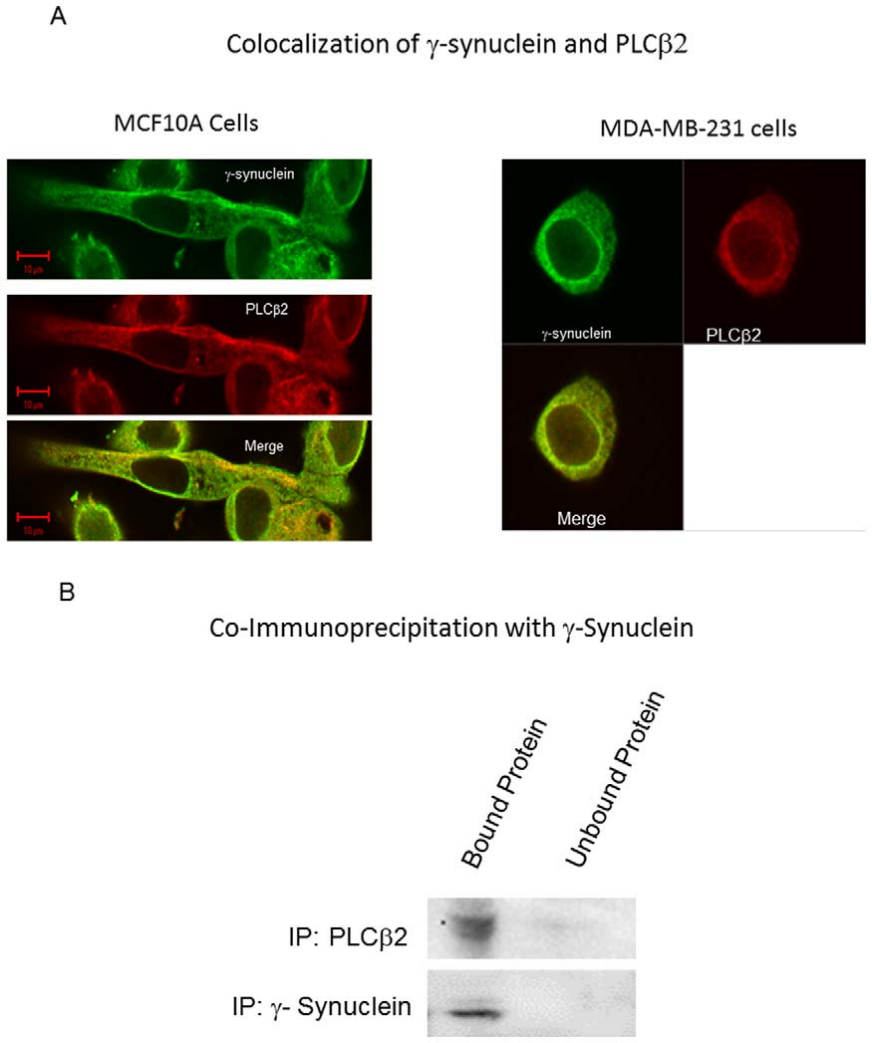
PLCβ2 and γ-synuclein associate in cells. **A** **–** Co-immunofluroescence studies showing the colocalization of γ-synuclein (green) and PLCβ2 (red) in two breast cancer cell lines, MCF10A and MDA-MB-231. **B-** Co-immunoprecipation of endogenous γ-synuclein and PLCβ2 in MDA-MB-231 cells.

As an initial step in understanding the relationship between γ-synuclein and PLCβ2 in breast cancer, we determined whether these proteins associate in cells. We first visualized their cellular localization and colocalization in two cultured breast cancer epithelial cell lines, MCF10A representing stage 1 cancer, and MDA-MB-231 representing stage 4 breast cancer (see [11]). As shown in **Fig. 1a**, the proteins are widely distributed through the plasma membrane and cytoplasm with a low nuclear population. Also, we find a moderate degree of colocalization of the endogenous proteins and a Mander's coefficient of 0.55±0.05 was obtained for the proteins in both cell lines. This value can be compared to a positive control of 0.93±0.01 (n = 9) measured for an eGFP-tagged protein that was immunostained with Alexa647, and a negative control of 0.017+0.02 (n = 7) measured for an eGFP-tagged protein with only Alexa647-secondary antibody (Calizo and Scarlata, submitted).

To corroborate the colocalization studies suggesting cellular association of γ-synuclein and PLCβ2, we carried out co-immunoprecipitation studies in MDA-MB231 cells. The results, shown in **Fig. 1b** **,** support the idea that these proteins associate in cells.

### *In vitro,* γ-synuclein binds to PLCβ2 with high affinity

We characterized the interaction between γ-synuclein and PLCβ2 by studying the association of these proteins in solution. For these studies, we covalently attached the fluorescent probe CPM onto purified PLCβ2 (see Methods). CPM is highly sensitive to the polarity of its environment and binding of a CPM-labeled protein to an unlabeled partner usually results in a large increase in CPM fluorescence. CPM is thiol-reactive and can attach to one of the many Cys side chains distributed throughout the catalytic and C-terminal domains along with one in the EF hand region. We have previously found labeling PLCβ2 with a thiol reactive probe does not affect its activity or G protein activation properties [22]. Addition of binding partners to CPM-PLCβ2 results in a concentration-dependent increase in fluorescence intensity that quantitatively gives identical affinities as FRET and qualitatively by other methods (see [22], [23]). We note that CPM is essentially non-fluorescent in its unreacted form, and although addition of excess protein to unreacted probe will increase its fluorescence, its signal never exceeded 1% of the signal obtained for any of the samples used in this study. We added freshly prepared and purified γ-synuclein to a solution of CPM- PLCβ2.

**Figure 2:**
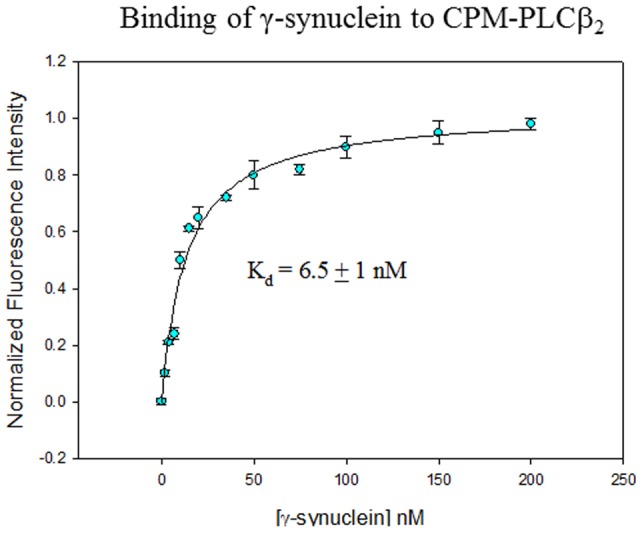
PLCβ2 and γ-synuclein associate in solution. Normalized change in fluorescence intensity of 2 nM CPM- PLCβ2in solution as purified γ-synuclein is added where the total intensity increase was 39±4%. The data shown are corrected for dilution and background, which was less than 1% of the signal, and are an average of 3 sets of measurements.

We observed a systematic increase in CPM-PLCβ2 intensity (i.e. 39±4%) when γ-synuclein was incrementally added. No changes in intensity were detected when dialysis buffer was substituted for γ-synuclein. An example of a fluorescence titration curve is shown in **Fig. 2** where the data were corrected for background, which was less than 1% of the signal, and also for dilution. Fitting the titration curve to a biomolecular dissociation constant gives a K_d_ = 6.5±1 nM. This same binding constant is obtained when the initial concentration of CPM- PLCβ2 was reduced from 2.0 to 0.5 nM (K_d_ = 7.8±2 nM) supporting the idea that we are viewing a protein-protein association. Substitution of the full length CPM-labeled enzyme with only its labeled N-terminal PH domain (CPM-PH-PLCß2) or with CPM-Rac1 (*see below*) gave a smaller increase in intensity at much higher protein concentrations suggesting that the affinities for these other proteins is at least 50 fold weaker, and indicating that we are indeed observing association of γ-synuclein with proteins and not with the CPM label itself.

### Identification of the binding region between γ-synuclein and PLCß2

**Figure 3:**
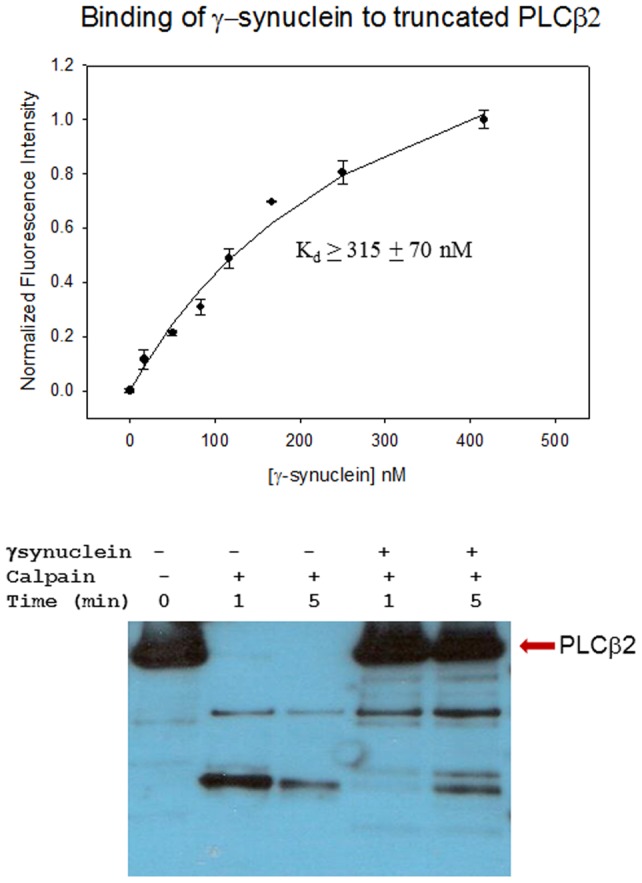
The C-terminus of PLCβ2is required for strong γ-synulcein binding. ***Top*** – Association of γ-synuclein to a C-terminal truncated PLCβ2 construct under identical conditions as in **Fig. 2**. While the affinity cannot be accurately obtained since the titration curve did not plateau, the estimated the minimum apparent dissociation constant is given. ***Bottom*** – Calpain digestion of PLCβ2 showing cleavage of the ∼134 kDa enzyme into ∼80 and ∼45 kDa fragments where identical amounts of PLCβ2 were loaded onto each lane. Note the protection of enzyme digestion in the presence of γ-synuclein.

We tested whether γ-synuclein would alter the interaction between PLCβ2 and its activators. Since different G protein subunits associate to different regions of the enzyme, we first set out to identify the PLCβ2 domain where γ-synuclein binds. Rac1 and Gßγ bind to the N-terminal PH domain of PLCβ2, and so we measured the association of γ-synuclein to a PLCβ2 construct that is missing the long ∼400 amino acid C-terminal region (see [19]). We find that γ-synuclein binds to this truncated enzyme at a far lower affinity than the whole enzyme (**Fig. 3** **top**). This result, along with the fluorescence titrations described above, suggests that γ-synuclein interacts primarily with the C-terminal region of the protein.

PLCβ2 has a calpain cleavage site that releases the entire C-terminal tail region and truncation can be seen by the shift in the 133.7 KDa enzyme electrophoresis band to ∼80 and ∼45 KDa bands (see [24]). In **Fig. 3** **bottom** we show that γ-synuclein protects PLCβ2 from calpain digestion. We extracted the 100 KDa band obtained from calpain-digested PLCβ2 and analyzed this product by mass spectrometry. Comparing this fragment to the undigested and γ-synuclein – PLCβ2 complex, we find that γ-synuclein binding protects PLCβ2 from calpain cleavage at residue 753. This residue lies in a region that connects the C2 domain with the C-terminal tail.

### γ-Synuclein inhibits Gαqbut not Gßγ association to PLCß2

**Figure 4:**
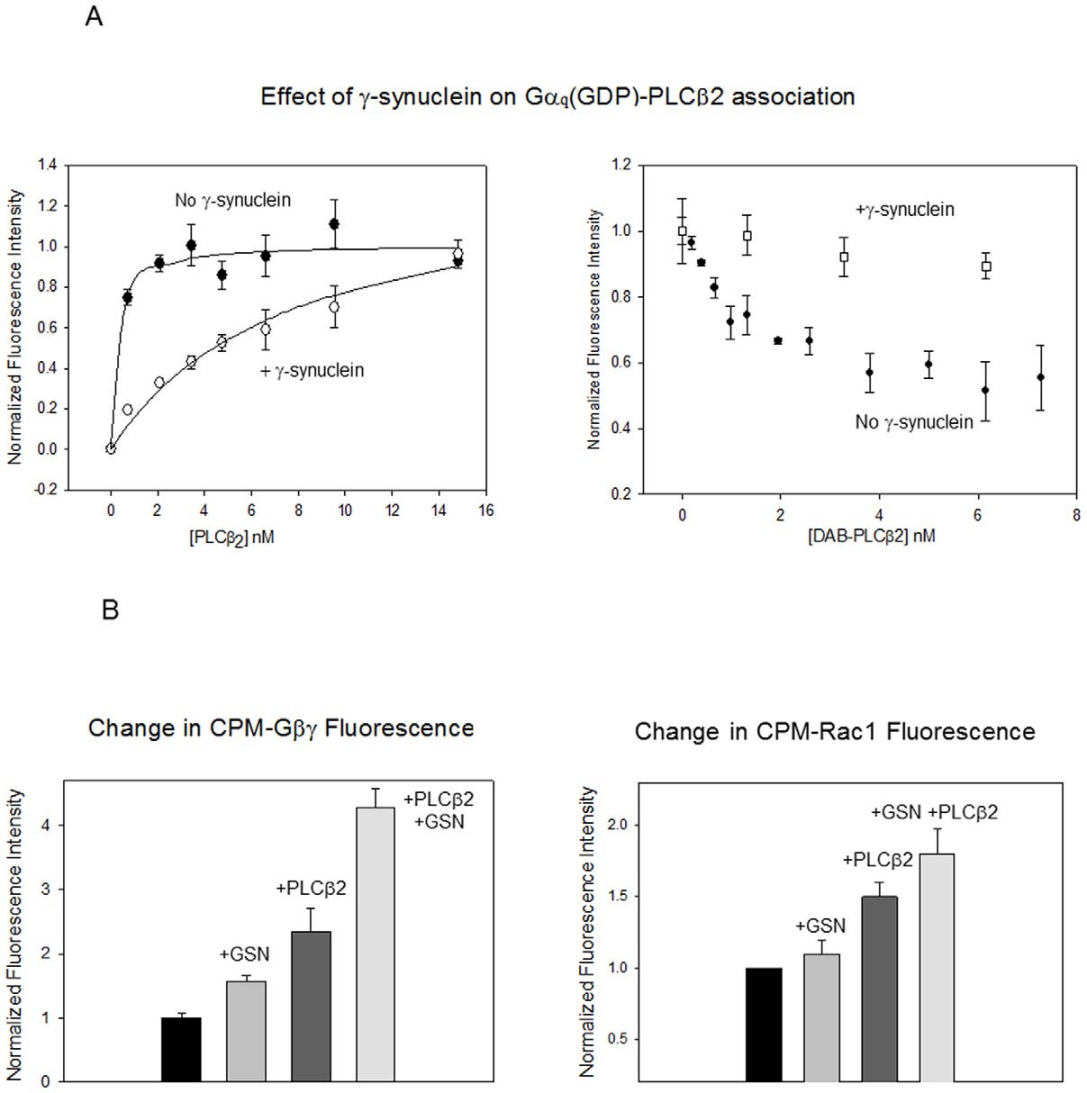
γ-Synuclein inhibits the binding of Gαqbut not Gßγ or Rac1, to PLCß2. ***A*** – *(left)* Fluorescence titrations showing that 20 nM γ-synuclein inhibits the association of PLCβ2 to 2 nM CPM-Gα_q_(GDP) as seen by the 27±3% increase in CPM-Gαq fluorescence as PLCβ2 is added (n = 4) and *(right)* by the ability of 20 nM γ-synuclein to prevent FRET when PLCβ2 with a non-fluorescent energy transfer acceptor (DAB) is added to CPM-Gαq(GDP), n = 3. ***B***– *(left)* Change in the intensity of 10 nM CPM-Gßγ when 100 nM γ-synuclein is added, when 10 nM PLCβ2 is added, and when 100 nM γ-synuclein and 10 nM PLCβ2 are added, n = 2. *(right)* Change in the intensity of 2 nM CPM-Rac1(GDP) when 5 nM PLCβ2is added, when 20 nM γ–synuclein is added and when 5 nM PLCβ2 and 20 nM γ-synuclein were added.

The studies above show that γ-synuclein binds to the region of PLCβ2 that is close to the binding region of Gαqas indicated by the recent crystal structure of a truncated PLCβ3 construct complexed with Gαq [25] and overlaps with the region required for Gαqactivation [26]. Thus, we tested whether γ-synuclein could compete with Gαqfor PLCβ2 association by two complementary types of fluorescence titrations (**Fig. 4A** **)**. In the first, we measured the association between CPM-Gα_q_(GDP) and PLCβ2 in the absence and presence of a ten-fold excess of γ -synuclein. In the second, we added PLCβ2 labeled with a non-fluorescent FRET acceptor (Dabcyl) to CPM- Gα_q_(GDP) and measured their association by the decrease in CPM donor fluorescence in the presence and absence of γ-synuclein. Both studies show a clear inhibition of Gα_q_(GDP) – PLCβ2 binding when γ-synuclein is present (**Fig. 4A** **)**. Activation of Gαqresults in an ∼20 fold increase in affinity for PLCβ2 [22], and we find that a ten-fold excess γ-synuclein has little, if any, affect on the association between activated Gαqand PLCß2, (K_d_ = 0.3±0.2 nM without γ-synuclein and 0.9±0.3 with γ-synuclein, p = 0.049).

We also determined whether γ-synuclein could affect Gßγ -PLCβ2 interactions. Although we could not detect changes in Gßγ-PLCβ2 association in the presence of excess γ-synuclein, we found evidence that γ-synuclein weakly associated to the Gßγ-PLCβ2 complex. When γ-synuclein was added to CPM- Gßγ, an increase in intensity was observed and a further increase occurred upon the addition of PLCβ2 (**Fig. 4B** **left**). An increase in intensity was also seen when γ-synuclein was added to the Gßγ-PLCβ2 complex. These results indicate that γ-synuclein may form a ternary complex with Gßγ- PLCβ2. Identical results were obtained with deactivated or activated Rac1 (**Fig. 4B** **right**). Thus, γ-synuclein may form ternary complexes with Gßγ-PLCβ2 and Rac1-PLCβ2 but not with Gαq-PLCβ2.

### γ-Synuclein inhibits the activity of PLCß2, but not its activation by G proteins

**Figure 5:**
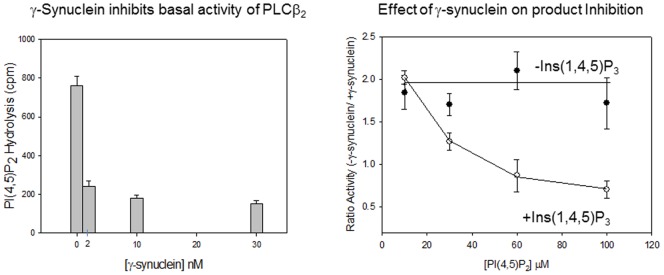
γ-Synuclein inhibits the enzymatic activity PLCβ2. *(left)* Change in 20 nM PLCβ2 activity with increasing amounts of γ-synuclein. (*right*) Ratio of PLCβ2 activity with increasing substrate in the presence and absence of 10 mM Ins(1,4,5)P_3_ showing that γ-synuclein promotes product inhibition.

We determined the impact of γ-synuclein on the activity of PLCβ2. These studies were carried out by measuring the changes in PLCβ2-catalyzed hydrolysis of ^3^H-PI(4,5)P_3_. We find that the presence of γ-synuclein decreases PLCβ2 activity approximately 4 fold (**Fig. 5**). This decrease is more pronounced than the decrease seen using α-synuclein [27].

Rac1 has been reported to activate PLCβ2 by increasing its affinity for lipid membranes [12] although this does not appear to be the case in purified systems (Golebiewska & Scarlata, *unpublished*). Nevertheless, we determined whether γ-synuclein affected PLCβ2 activity by altering its membrane interactions. These studies were done by measuring changes in binding of PLCβ2 to large, unilamellar vesicles composed of PC/PS/PE at a 1∶1∶1 molar ratio in the presence and absence of excess γ-synuclein. Membrane binding was monitored by the large increase in fluorescence intensity of CPM- PLCβ2 that occurs when the enzyme binds to membranes (see [28]). We find that a fourfold excess of γ-synuclein (100 nM) does not affect the partition coefficient for membrane binding of PLCβ2 (45±10 µM, n = 4 without γ-synuclein versus 39±18 µM, n = 3 with γ-synuclein). These data suggest that PLCβ2 binds to membranes with a similar affinity as the PLCβ2- γ-synuclein complex.

We have previously found that Gßγ activates PLCβ2 by increasing the rate of release of Ins(1,4,5)P_3_ product [21]. With this in mind, we determined whether γ-synuclein inhibits PLCβ2 by inhibiting the release of product. We measured product inhibition of PLCβ2 and the PLCβ2- γ-synuclein complex as a function of substrate (PI(4,5)P_2_) concentration. In the absence of product, γ-synuclein did not affect the change in PLCβ2 with increasing amount of substrate. However, in the presence of γ-synuclein, we find that Ins(1,4,5)P_3_ reduces the ability of PI(4,5)P_2_ to promote activity (**Fig. 5B**). Our interpretation of these results is that γ-synuclein stabilizes an early or intermediate conformation of the enzyme where product is bound more strongly in the active site.

### G proteins reverse γ-synuclein inhibition of PLCß2

**Figure 6:**
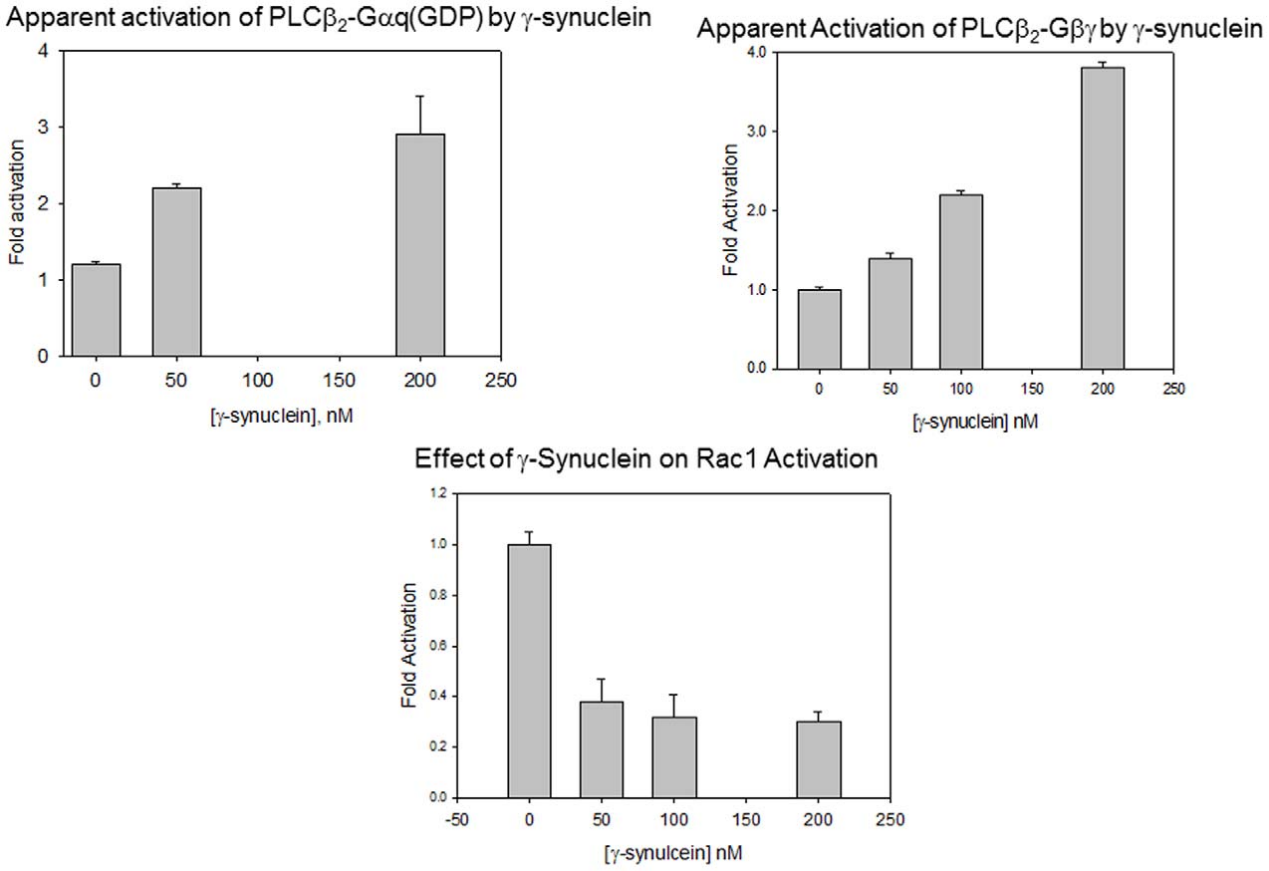
γ-Synuclein binding to PLCβ2 results in an apparent increase in G protein activation. ***Top panels*** – Apparent activation of 20 nM PLCß2, as calculated by the ratio of the activity of PLCβ2 complexed with Gαq(*left*) or Gßγ (*right*) over PLCβ2 alone, due to reversal of γ-synuclein by G protein subunits. ***Bottom*** – Activity studies showing that Rac1 does not interfere with inhibition of PLCβ2.

Our binding studies predict that γ-synuclein should effect activation of PLCβ2 by Gαqby direct competition, but not by Gßγ or Rac1. We first tested this idea by measuring the ability of γ-synuclein to diminish activation of PLCβ2 by Gα_q_. We found that addition of an 80 fold excess of γ-synuclein did not reduce the activity of the PLCß2-Gαqcomplex, due to the very strong affinity between PLCβ2 and activated Gα_q_. However, because γ-synuclein inhibits PLCβ2, the ratio of Gαq/PLCβ2/γ-synuclein activity versus γ-synuclein -PLCβ2 shows an apparent activation (**Fig. 6**). This apparent activation is due to the ability of Gα_q_(GTPγS) to displace γ-synuclein from PLCβ2 and cause a reversal in γ-synuclein inhibition as well as activation.

We also determined whether γ-synuclein could affect activation of PLCβ2 by Gßγ and Rac1. We found that even though γ-synuclein inhibits isolated PLCβ2, it does not inhibit the PLCβ2- Gßγ complex. In fact, the addition of Gßγ to PLCβ2 overcomes γ-synuclein inhibition so that when the ratios of PLCβ2- γ-synuclein/PLCβ2 as a function of γ-synuclein are plotted, an apparent activation is seen (**Fig. 6**). In contrast, γ-synuclein similarly inhibits PLCβ2 and PLCβ2-Rac1 in accord with the idea that Rac1 may enhance activity by promoting membrane interactions of the enzyme [14].

## Discussion

In this study, we have established a link between γ-synuclein expression and PLCβ2 activation in cultured cells. While the function of PLCβ2 is to transform G protein signals into calcium responses [29], [30], little is known about the cellular function of γ-synuclein in neuronal cells, where it is highly expressed, or in certain transformed cells (but see http://www.disprot.org/protein.php?id=DP00630). A large over-production of γ-synuclein in breast cancer was first noted by Shi and coworkers who identified it as the breast cancer specific gene protein 1, which was later found to be γ-synuclein [7]. Anticancer agents that target γ-synuclein have been designed [31]. Over-production of PLCβ2 correlates with the severity of breast cancer [10], [11]. Since PLCβ2 mediates mitogenic, proliferative and migratory events through its interactions with heterotrimeric and monomeric G proteins, we reasoned that γ-synuclein might not only bind PLCβ2 and affect its basal activity, but also affect G protein activation.

γ-Synuclein is classified as an unstructured protein and has several potential binding partners (see [32]). Thus, we first determined whether PLCβ2 is a natural binding partner of γ-synuclein in cultured cells. In these studies, we visualized the two proteins by immunofluorescence in MCF 10A and MDA MB231 cells that mimic stage 1 and 4 breast carcinoma, respectively. We find a high degree of colocalization in both cell lines, and additionally, the protein coimmunoprecipate. Thus, γ-synuclein appears to be a cellular binding partner of PLCβ2.

We have previously carried out studies that investigated the interaction between another PLCβ family member, PLCβ1, and α-synuclein [24]. Both proteins are highly expressed in neural tissue. We found that the region of interaction between these proteins is the same and that their cellular association prevents calcium-stimulated degradation of the enzyme by the protease calpain. While it is unclear whether calpain degradation plays an important role in cells expressing γ-synuclein, it is probable that γ-synuclein will also protect PLCβ2 from cleavage. This stabilization could underlie the correlation between elevated PLCβ2 levels with increased γ-synuclein expression making it difficult to quantify the decrease in calcium release through PLCß with increased cellular γ-synuclein (Yerramilli et al., unpublished).

We characterized the affinity of γ-synuclein-PLCβ2 association using purified proteins. Fluorescence studies show that the two proteins associate strongly in solution with an affinity that is slightly stronger than Gα_q_(GDP), Gßγ and Rac1 but much weaker than activated Gα_q_. Interestingly, the γ-synuclein binding site suggested by the calpain digestion studies overlaps with the Gαqbinding site. We speculate that when γ-synuclein expression is very high, as in many carcinomas, PLCβ2-γ-synuclein may disrupt preformed PLCβ2-Gα_q_-receptor complexes in the basal state (see [33], [34]) or perturb the cellular localization of the enzyme. However, upon activation, it is likely that Gαqcan displace γ-synuclein from PLCβ2 allowing γ-synuclein to homo- or hetero-oligomerize with other proteins. Importantly, activation of PLCβ2 by G proteins to promote mobility and migration is preserved in the presence of γ-synuclein.

Our studies suggest that γ-synuclein binding decreases the basal activity of PLCβ2 through a mechanism that appears to involve product release rather than access to substrate by inhibition of membrane interactions. Since Gαqbinds to the same region of the PLCβ2 as γ-synuclein, it is not surprising that it reverses γ-synuclein inhibition by direct displacement. In contrast, Rac1 associates to the extreme N-terminus [35] and we find that this association does not perturb γ-synuclein binding or inhibition. In contrast, Gßγ interacts with both the N-terminal pleckstrin homology domain and the catalytic domain (see [13]) and can reverse inhibition of PLCβ2 by γ-synuclein. This reversal is not due to competition of γ-synuclein binding by Gßγ since γ-synuclein binds well to both PLCβ2 and PLCβ2-Gßγ. Instead, our data suggest that ternary complexes can form (**Fig. 4**). We hypothesize that the mechanism through which Gßγ activates the enzyme does not allow for γ-synuclein inhibition. Since Gßγ and γ-synuclein inversely affect product release, (**Fig. 5** and [36]), it is possible that the conformational changes associated with product release which are promoted by Gßγ are preserved in the presence of γ-synuclein, and that displacement of γ-synuclein may not be required for reversal of inhibition. Thus, Gßγ is a more potent activator of PLCβ2- γ-synuclein than isolated PLCβ2. More studies are needed to understand the conformational changes associated with product release. Regardless of the mechanism, inhibition of PLCβ2 by γ-synuclein may not have significant cellular effects since the basal activity of PLCβ2 is low. However, the ability of Gαqand Gßγ to activate PLCβ2 while simultaneously reversing inhibition may lead to an apparently more robust calcium signals.
